# Catalogue of antibiotic resistome and host-tracking in drinking water deciphered by a large scale survey

**DOI:** 10.1186/s40168-017-0369-0

**Published:** 2017-11-28

**Authors:** Liping Ma, Bing Li, Xiao-Tao Jiang, Yu-Lin Wang, Yu Xia, An-Dong Li, Tong Zhang

**Affiliations:** 1 Environmental Biotechnology Laboratory, The University of Hong Kong, Pokfulam Road, Hong Kong, China; 2 Graduate School at Shenzhen, Tsinghua University, Shenzhen, China

**Keywords:** Drinking water, Public health, Antibiotic resistome, Host-tracking, Horizontal gene transfer, Bacterial community

## Abstract

**Background:**

Excesses of antibiotic resistance genes (ARGs), which are regarded as emerging environmental pollutants, have been observed in various environments. The incidence of ARGs in drinking water causes potential risks to human health and receives more attention from the public. However, ARGs harbored in drinking water remain largely unexplored. In this study, we aimed at establishing an antibiotic resistome catalogue in drinking water samples from a wide range of regions and to explore the potential hosts of ARGs.

**Results:**

A catalogue of antibiotic resistome in drinking water was established, and the host-tracking of ARGs was conducted through a large-scale survey using metagenomic approach. The drinking water samples were collected at the point of use in 25 cities in mainland China, Hong Kong, Macau, Taiwan, South Africa, Singapore and the USA. In total, 181 ARG subtypes belonging to 16 ARG types were detected with an abundance range of 2.8 × 10^−2^ to 4.2 × 10^−1^ copies of ARG per cell. The highest abundance was found in northern China (Henan Province). Bacitracin, multidrug, aminoglycoside, sulfonamide, and beta-lactam resistance genes were dominant in drinking water. Of the drinking water samples tested, 84% had a higher ARG abundance than typical environmental ecosystems of sediment and soil. Metagenomic assembly-based host-tracking analysis identified *Acidovorax*, *Acinetobacter*, *Aeromonas*, *Methylobacterium*, *Methyloversatilis*, *Mycobacterium*, *Polaromonas*, and *Pseudomonas* as the hosts of ARGs. Moreover, potential horizontal transfer of ARGs in drinking water systems was proposed by network and Procrustes analyses.

**Conclusions:**

The antibiotic resistome catalogue compiled using a large-scale survey provides a useful reference for future studies on the global surveillance and risk management of ARGs in drinking water.

**Graphical abstract:**

.
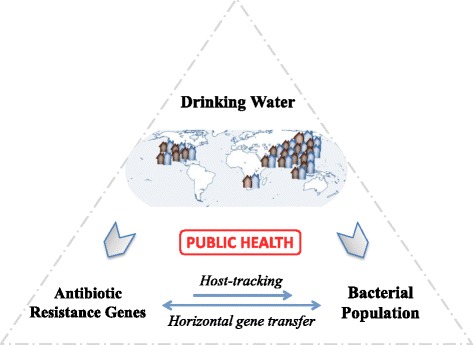

**Electronic supplementary material:**

The online version of this article (10.1186/s40168-017-0369-0) contains supplementary material, which is available to authorized users.

## Background

The overuse and misuse of antibiotics, not only for human therapy, but also for livestock breeding around the world over the past decades have led to the emergence and excess of antibiotic resistance genes (ARGs) and antibiotic resistant bacteria (ARB) in a diverse range of environments [1–4]. Among the various ARB and ARG reservoirs, aquatic ecosystems are considered to be the most important due to the high mobility of organisms and genetic elements [5]. ARGs have been reported to rapidly spread, conducted by mobile genetic elements in ecosystems [6]. The rapid transfer and spread of ARGs among bacterial cells [7] could be facilitated by mobile genetic elements, including plasmids, transposons and integrons, etc. Recently, findings on ARGs in drinking water distribution systems, especially in treated drinking water that may have direct contact with human beings, have given rise to medical concerns from both researchers and the public [8–10]. Although most microorganisms can be effectively removed after the treatment process, disinfection resistant microbes can proliferate in the drinking water distribution system [10]. Previous studies have reported that drinking water chlorination could contribute to the enrichment of ARGs, likely induced by the underlying mechanisms of cross- or co-resistance to disinfectants and antibiotics [9, 11]. Thus, disinfection resistant microorganisms may carry more ARGs after drinking water treatment, causing potential risks and deserving more public attention.

To date, the molecular study of the microbial community and ARGs in drinking water at the user end has been mainly restricted by the following difficulties: (1) low biomass concentration for DNA extraction, (2) sample collection logistics, and (3) sampling standardization. Because of these difficulties, few studies have been conducted regarding the spatial variations of ARGs and microbial communities in drinking water at the point of use. However, the occurrence of ARGs in the point of use of drinking water may pose direct threats to human health and deserves more attentions from the public. Forsberg et al. found that soil bacteria structures resistomes across habitats, indicating the horizontal gene transfer (HGT) of ARGs between soil bacteria was in low frequency, in contrast to human pathogens [12]. Whether drinking water bacteria drive resistomes and the potential risks of HGT both remain largely unknown. The in-depth investigation of ARGs and bacterial community profiles in large-scale drinking water samples is central to understanding the overall picture, which is essential for decision-making about water management to control antibiotic resistance in drinking water systems.

In the present study, we collected drinking water samples at the point of use from 25 cities in seven countries and regions, including mainland China, Hong Kong, Macau, Taiwan, South Africa, Singapore, and the USA. We applied a metagenomic approach to achieve the following goals: (1) detect the antibiotic resistome in drinking water samples over a wide range of regions, (2) investigate the correlation between bacteria and resistomes, and (3) explore the potential hosts of ARGs. This large sequencing data set for a wide scope survey on the microbial community and ARGs in drinking water reflects the comprehensive resistome profiles and reveals the potential risks to human health caused by ARGs in drinking water.

## Methods

### Tap water sampling, pretreatment, and DNA extraction

Drinking water samples were collected from the point of use of 25 cities in mainland China (*n* = 20), Hong Kong (*n* = 1), Macau (*n* = 1), South Africa (*n* = 1), Singapore (*n* = 1), and the USA (*n* = 1). Additional sample descriptions can be found in supporting information, Additional file 1 Table S1 and Additional file 1 Figure S1. High-performance cartridge-type water purifiers (Torayvino, Toray Industries Inc., Japan) were installed on taps according to a protocol described elsewhere [8], to collect microorganisms by filtering tap water. Approximately 2000 L of tap water were filtered, controlling the flow rate of ~ 40 L/h, for about 48 h. After filtration, the purifiers were immersed into 50% ethanol solution to fix the captured microorganisms. The filters were delivered back to the laboratory within 72 h using an ice box. Upon arrival, the hollow fiber filter within purifiers was taken out and immersed into 100 mL ultrapure water and then treated using ultrasonication (Branson Ultrasonics Corp., USA) for 15 min to detach the microbial cells. The cells in water were subsequently collected by the filtration using a 0.45-μm cellulose ester membrane (Millipore Corp., USA). The effectiveness of capturing microorganisms by this approach had been evaluated before [8]. The membranes were stored at − 20 °C before DNA extraction. Additionally, three water purifiers with no tap water filtration were used as blank samples. Genomic DNA was extracted using FastDNA SPIN Kit for Soil (MP Biomedicals, France) following the standard protocol. DNA concentration was measured by Qubit^®^ 2.0 Fluorometer (Invitrogen, Life techniques). The DNA concentration of the three blank samples was below the detection limit.

### Metagenomic sequencing

DNA for each sample (5 μg) was used for 350 bp library construction (Nextera^®^ DNA Library Preparation Kit), and paired-end (2 × 100 bp reads) metagenomic sequencing was performed on an Illumina HiSeq 4000 platform in the Beijing Genomics Institute (BGI). Data filtration was performed to guarantee the quality of the downstream analysis (Additional file 1 S1). Filtered data obtained from tap water samples was 120 Gb (giga base pairs) in total, which is the largest sequence data set reported to date on the study of ARGs in drinking water samples. The metagenomics data was deposited into the National Center for Biotechnology Information Short Reads Archive database (NCBI SRA) under the BioProject PRJNA305188.

### Illumina MiSeq sequencing for 16S rRNA genes

The V4 region (~ 265 nucleotides) of the 16S rRNA gene sequences was amplified using F515 (5′-GTGCCAGCMGCCGCGGTAA-3′) and R806 (5′-GGACTACHVGGGTWTCTAAT-3′) primers (Additional file 1 Table S2). Dual-index sequencing strategy for primers (adapter + barcode + pad + linker + primer; Additional file 1 Table S3) and reaction conditions used in this study have been described elsewhere [13]. PCR assays were performed in triplicate to avoid the variations during amplification, and purified PCR amplicons were pooled together and sequenced on Illumina MiSeq PE250 (BGI). All the drinking water samples were successfully amplified and generated 32,525–147,248 sequencing reads. Additionally, three water purifiers with no tap water filtration were used as blank samples. Two samples were selected to evaluate the biases of using primers with different barcodes. One sample was selected using different DNA extraction kits to assess the effects of the kits on DNA extractions (Additional file 1 S2). All 16S rRNA gene sequences generated from tap water samples were deposited into NCBI SRA under the BioProject PRJNA305188. Sequences were analyzed using Mothur software as described previously [13]. Sequencing depth was normalized to 30,000 sequences for each sample. 16S rRNA gene sequences were clustered into OTUs based on the similarity threshold of 0.97.

### Identification of ARG-like sequences

All the metagenomic sequencing data was searched for ARGs against a structured non-redundant ARDB database [14, 15] using Usearch + BLASTX with an E-value ≤ 10^−5^. A sequence (100 bp short reads) was designated as an ARG-like fragment if its best BLASTX alignment to reference ARG sequence showed a similarity of ≥ 90% and the alignment length was ≥ 25 amino acids [14]. This identification approach had been validated to have a high accuracy of ≥ 99.5% [16]. A package of customized scripts was developed for automatic classification of identified ARG-like sequences into 25 “ARG types” (e.g., aminoglycoside resistance gene) and 619 “ARG subtypes” (e.g., *aad*A, *aad*B, etc.) [14]. To assess the ARG distributions in tap water samples, the abundance of ARGs was normalized and expressed as “copy of ARG per cell” (*capc*) using the following equation [17]:

$$ \mathsf{Abundance}={\sum}_{\mathit{\mathsf{i}}}^{\mathit{\mathsf{n}}}\frac{{\mathit{\mathsf{N}}}_{\mathit{\mathsf{i}}\;\left(\mathsf{ARG}-\mathsf{like}\mathsf{s}\mathsf{equence}\right)}\times \mathsf{100}/{\mathit{\mathsf{L}}}_{\mathit{\mathsf{i}}\;\left(\mathsf{ARG}\mathsf{s}\mathsf{reference}\mathsf{s}\mathsf{equence}\right)}\;}{{\mathit{\mathsf{N}}}_{\mathsf{16}\mathsf{S}\;\mathsf{s}\mathsf{equence}}\times \mathsf{100}/{\mathit{\mathsf{L}}}_{\mathsf{16}\mathit{\mathsf{S}}\;\mathsf{s}\mathsf{equence}}}\times {\mathit{\mathsf{N}}}_{\mathsf{16}\mathit{\mathsf{S}}\;\mathsf{copy}\mathsf{number}} $$

where *N*_*i* (ARG-like sequence)_ is the number of the ARG-like reads annotated as one specific ARG reference sequence, *L*_*i* (ARGs reference sequence)_ is the sequence length (bp) of the corresponding ARG reference sequence, *N*_16S sequence_ is the number of the 16S rRNA gene sequence identified for the metagenomic sequencing data by comparison to Greengenes database [16], *L*_16S sequence_ is the average length of 16S rRNA genes (1432 bp) in Greengenes database [18], *n* is the number of mapped ARG reference sequences belonging to that ARG type or subtype, 100 is the sequence length (bp) of the metegenomic reads, and *N*_16S copy number_ is the average copy number of 16S rRNA genes per cell. The average copy number within cells was calculated using CopyRighter [19] based on the “.biom” file generated by QIIME. A clustering analysis (CA) and a Principal Coordinates Analysis (PCoA) was performed based on the abundance matrix of ARG subtypes of all the tap water samples, using PAleontological STatistics software (PAST, version 3.09).

### Metagenomic assembly and identification of ARG-carrying contigs

After quality control, the metagenomic sequences were assembled using IDBA algorithm (version 1.1.1) [20]. The identification of ARG-carrying contigs (ACCs) was conducted following the strategy proposed in our previous study [7]. In brief, contigs were assembled using IDBA with default parameters. The open reading frames (ORFs) prediction was conducted using Prodigal (version 2) [6]. Then, the predicted ORF sequences were searched against structured non-redundant ARDB database for ARG-like ORFs identification using BLASTX under an E-value ≤ 10^−10^ [21]. An ORF sequence was considered to be an ARG-like ORF if its best BLASTX hit alignment to ARG sequences was under a cutoff of ≥ 80% similarity and ≥ 70% query coverage [21]. The identified ARG-like ORFs were then classified according to the structured non-redundant ARDB database.

### Taxonomic annotation of ARG-carrying contigs

To perform the taxonomic annotation of the identified ACCs, the ORF sequences of each ACC were compared to the local NCBI NR database using BLASTP with an E-value ≤ 10^−5^ [22] and then were parsed and annotated using MEGAN (MEtaGenome ANalyzer, version 5) [23]. An in house R script was used to assign taxa to contigs. In short, if more than 50% of the ORFs on a contig were attributed to the same kingdom/phylum/class/order/family/genus, then the contig was assigned to that taxon [24].

### Network analysis

Previously, network analysis was extensively used for the exploration of the underlying associations among genes, proteins, and microorganisms in complex microbial communities [25, 26]. In the present study, a correlation matrix was constructed with ARG and 16S rRNA data to explore the potential correlations of ARG–ARG, ARG–bacteria, and bacteria–bacteria by calculating all pairwise Spearman’s correlation coefficients (*ρ*) among ARG subtypes that occurred in at least 40% of the tap water samples. A correlation between two nodes was regarded as statistically significant for *ρ* ≥ 0.6 and *P* value ≤ 0.01. *ρ* and *P* value were generated via R-function “rcorr” (Hmisc package). To reduce the frequency of false-positive results, the *P* values were then adjusted using Benjamini–Hochberg method [27]. The strong pairwise correlations among ARGs and species abundances formed correlation networks. The network analysis was performed in R environment using igraph, VEGAN, and Hmisc packages, and was visualized by the interactive platform of Gephi (version 0.9.0).

### Procrustes analysis

To assess the potential for HGT of ARGs in drinking water, Procrustes analysis was performed based on the previously published hypothesis that bacterial phylogeny may structure the resistome (HGT in low frequency) if there were a strong correlation between the ARG profile and bacterial composition [12]. In brief, the difference between groups (ARG-bacteria) was analyzed using a one-way ANOVA test with Tukey post-hoc tests. A *P* value of < 0.05 (two-sided) was considered as statistically significant. Procrustes transformations were performed using two Bray-Curtis distances plots (PCoA) as input based on the matrix of microbial community and ARGs at subtype level. The measure of fit *M* ^2^ (the sum of squared distances between matched sample pairs) and *P* values were determined from 10,000 labeled permutations [12].

## Results and discussion

### Broad-spectrum profile of ARG abundance in tap water

**Fig. 1:**
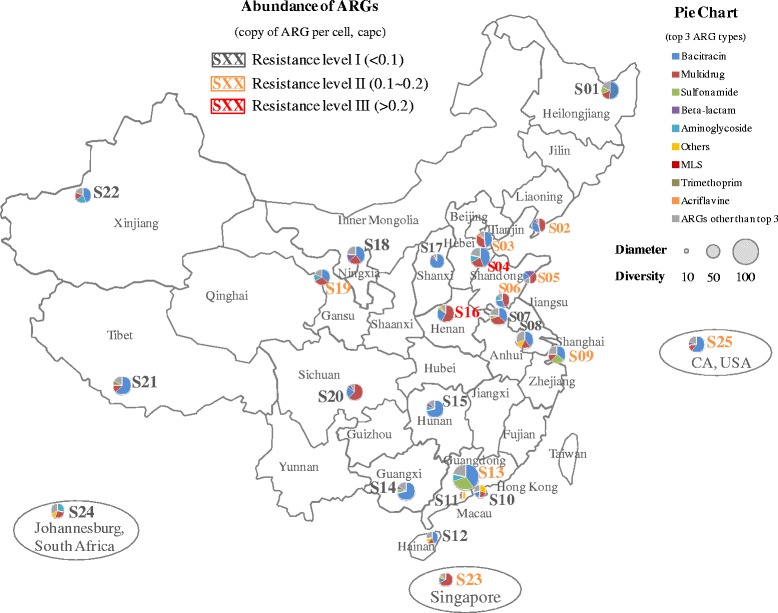
The abundance and diversity of ARGs in drinking water samples. a. Color of sample ID shows the resistance level of ARGs (I, II and III). b Pie chart presents the profiles of ARG abundance (top 3 ARG types). c Diameter of pie chart indicates the ARG diversity (number of ARG subtypes). *MLS: Macrolidelincosamide-streptogramin*

In total, 181 ARG subtypes belonging to 16 ARG types were detected in at least one of the 25 tap water samples (Additional file 1 Table S4 and Additional file 1 Table S5). The ARG diversity (number of ARG subtypes) was in the range from 16 (sample S11, Macau, China) to 88 (sample S13, Guangdong province, China; Fig. 1). Overall, 14 tap water samples belonged to resistance level I (< 0.1 *capc*), 9 belonged to resistance level II (0.1~0.2 *capc*), and 2 belonged to resistance level III (> 0.2 *capc*; Additional file 1 Table S6). The highest ARG enrichment (resistance level III) was detected in samples S16 (4.3 × 10^−1^ *capc*, Henan province) and S04 (3.7 × 10^−1^ *capc*, Hebei province), which were both collected from northern China. This ARG enrichment was ~ 12-fold higher than that of the lowest S12 (Hainan province, southern China). Interestingly, Zhang et al. observed significantly high antibiotic emissions in the same regions, Henan and Hebei province of mainland China [28]. It has been shown previously that antibiotics released into the environment could contribute to the selection for antibiotic resistance [29]. The ARGs encoding resistance to bacitracin, multidrug, sulfonamide, beta-lactam, and aminoglycoside were more frequently detected in these tap water samples (Additional file 1 Table S7). As expected, these dominant ARGs were usually associated with antibiotics that have been extensively used in human or veterinary therapy [16]. Moreover, a moderate ARG level was observed in the drinking water of Singapore and the USA where tap water is considered to be direct potable water, and the similar ARG composition of their ARG compositions was revealed by cluster analysis (Additional file 1 Figure S2).

**Fig. 2:**
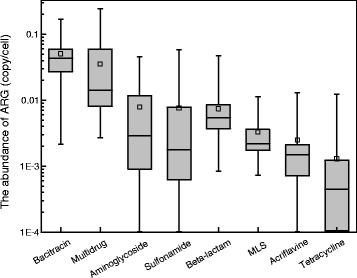
Abundance of the top 8 ARG types in the drinking water samples. Zero abundances were considered in the plot. *MLS: Macrolide-lincosamide-streptogramin*

We also compared the top 8 ARG types in the drinking water samples (Fig. 2). Bacitracin-ARGs were found to be the most dominant in 68% of the tap water samples, with an abundance of 2.2 × 10^−3^–1.7 × 10^−1^ *capc.* Multidrug-ARGs, which encode resistance to multiple antimicrobial drugs, dominated in 20% of the tap water samples with an abundance of 2.7 × 10^−3^–2.4 × 10^−1^ *capc*. In contrast with previously reported data showing that the dominant ARG types in sewage, animal feces, and activated sludge were multidrug-ARGs, tetracycline-ARGs, and aminoglycoside-ARGs, respectively [16], we found that bacitracin-ARGs had the highest abundance in drinking water. A few previous studies also reported the prevalence of bacitracin-ARGs in freshwater [30–32]. Bacitracin resistance genes were once considered to be intrinsic to bacteria as they are widespread in 153 genera [21]. Diverse bacitracin resistant strains were isolated from deep glacial ice, such as *Herminiimonas glaciei* [30] and *Dyadobacter hamtensis* [31]. All 60 *Aeromonas* strains isolated from freshwater in India were resistant to bacitracin [32]. Moreover, significantly high levels of bacitracin-ARGs were observed in treated wastewater and Tibetan sediment, with levels up to 70% of total ARG abundance [6, 33]. Bacitracin is used for topical treatment of localized skin lesions, eye infections, and also for the prevention of wound infections. Additionally, its application in chicken feed has been approved by the US Food and Drug Administration, which could be greatly contributing to bacterial antibiotic resistance. Although bacitracin-ARGs were detected in these drinking water samples, whether these resistance genes could bring potential risks to public health still requires more systematic researches.

**Fig. 3:**
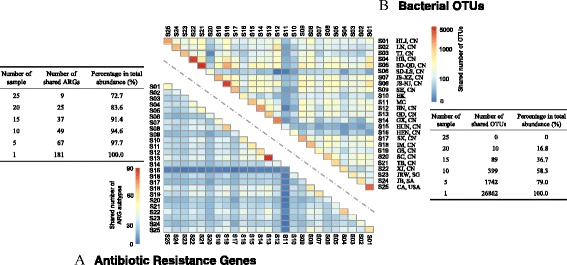
Shared ARG subtypes and taxa of drinking water samples. The *color scale* reflects the degree of **a** antibiotic resistance genes subtypes or **b** bacterial OTUs that are shared between the drinking water samples listed on the *horizontal axis* and along the *vertical axis.* Details of sample ID and abbreviation of sampling location are shown in Additional file 1 Table S1. For example, S01-HLJ, CN represents that drinking water S01 was collected from Heilongjiang province of China. The table shows the number of shared ARGs or bacterial OTUs and corresponding percentage of the shared ARGs or bacterial OTUs in total abundance

Among the 181 identified ARG subtypes, some were generalists existing in all samples (Additional file 1 Figure S3), for example, multidrug efflux protein, multidrug HAE1-family protein, multidrug *mex*F, beta-lactam TEM-2, macrolide *mac*B, and beta-lactam TEM-15, etc. Their abundance accounted for 72.7% of the total ARGs identified from the drinking water samples (Fig. 3). In total, 65 ARG subtypes were specialists only detected in one tap water sample, for example, the chloramphenicol resistance gene *flo*R was only detected in S04 (Hebei, China). Some of the ARG subtypes identified in this study were detected previously using traditional PCR-based techniques, such as *amp*C and *mec*A in municipal wastewater [34]; *tet*O, *tet*W, and *tet*Q in lagoons [35]; and *sul*I, *sul*II, and *bla*TEM in river and drinking water sources [36]. However, many of the identified ARG subtypes had not previously been revealed in water environments. Thus, the traditional PCR-based methods, limited by primers, could not provide comprehensive ARG profiles for drinking water samples.

To further explore the potential correlation among ARGs, network analysis was used. It revealed the ARG combinations of *mex*E-*mex*F-*opr*N and *sul*1-*aad*A-*aad*B in drinking water (modularity = 0.493; Additional file 1 Figure S4, Additional file 1 S3). These gene combinations were previously discovered on the whole genome of *Pseudomonas aeruginosa* and *Salmonella enterica*, respectively [37, 38]. Thus, the metagenomic based approach largely facilitated ARGs investigation over a larger spectrum without PCR bias and captured a more comprehensive picture of the correlation among ARG profiles in drinking water.

### Comparison of ARG profiles from drinking water and other environmental samples

**Fig. 4:**
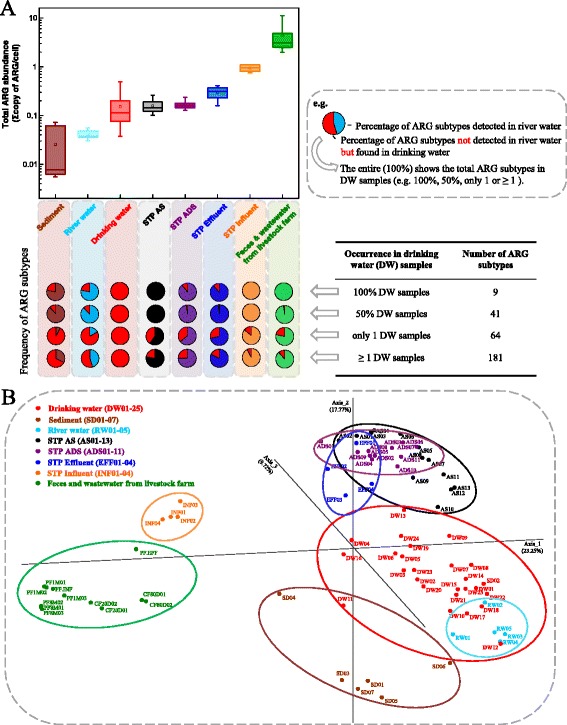
Comparison of ARGs in drinking water to other environments. **a** Comparison of the total ARG abundance in different environmental samples, and the frequency of ARG subtypes occurrence in drinking water, compared to other environmental samples. STP: sewage treatment plant; AS: activated sludge; ADS: anaerobic digestion sludge. **b** Principal Coordinate Analysis (PCoA) plot showing the ARG composition differences among the 81 environmental samples from 8 ecosystems (Bray-Curtis)

To explore ARGs autochthonous to drinking water samples, the catalogue of ARGs in drinking water was further compared to 56 environmental samples from seven niches, i.e., sediment (*n* = 7), river water (*n* = 5), sewage (*n* = 4), treated wastewater (*n* = 4), activated sludge (AS, *n* = 13), anaerobic digestion sludge (ADS, *n* = 11), and feces and wastewater from livestock farm (*n* = 12). Basic information about the 56 environmental samples is summarized in the supporting information (Additional file 1 Table S9), for which 39 metagenomic data sets have been used in our previous studies on the antibiotic resistome [7, 16]. In total, 501 ARG subtypes belonging to 20 ARG types were detected in the 81 environmental samples. The abundance of ARGs followed the order of sediment < river water < drinking water < sewage treatment plant (STP) ADS < STP AS < STP effluent < STP influent < feces and wastewater from livestock farm (Fig. 4a). In total, 84% of drinking water samples had higher total ARG abundance than that in sediment and soil, and 8% of samples (S04 and S16) had more ARGs than STP AS and STP ADS. The ARG levels in sewage and feces and wastewater from livestock farm were within 1–2 orders of magnitude higher than drinking water. The 181 identified ARG subtypes in the drinking water samples were detected in other environmental ecosystems, with a percentage of 32–92% (Fig. 4a). The nine ARG subtypes prevalent in all the drinking water samples were also found in activated sludge, influent, and livestock farm samples; however, two subtypes (Beta-lactam TEM-2 and TEM-15) were absent from sediment, river water, STP ADS, and STP effluent (Fig. 4a). The beta-lactam resistance genes of TEM-2 and TEM-15 were only prevalent in 9–25% of AS, ADS, and effluent samples but occurred in all STP influent samples (Additional file 1 Table S10). The grouping patterns shown in the PCoA plot demonstrated that drinking water samples were clearly separated from sewage and feces and clustered more closely with river water (Fig. 4b), indicated by a higher similarity of ARG profiles with natural water environments.

### The host of ARGs in tap water samples

**Fig. 5:**
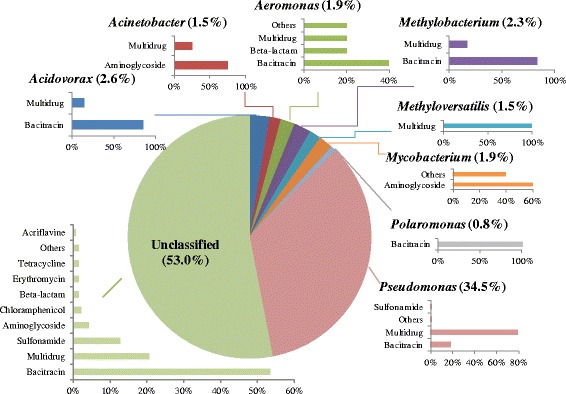
The taxonomy of ARG-carrying contigs (in genus level) and the percentages of ARG types these contigs carried. a Pie chart shows the taxonomy and percentage of ARG-carrying contigs. For example, *Pseudomonas* (34.5%) represents that 34.5% of ARG-carrying contigs were annotated as *Pseudomonas*. b Bar chart shows the percentages of ARGs types that were carried by the annotated ARG-carrying contigs. The percentage of these annotated ARG-carrying contigs was set as 100%. For example, 80% of the ARG-carrying contigs originating from *Pseudomonas* spp. carried multidrug resistance genes

There were 264 contigs assembled from drinking water metagenomes that carried ARGs. These ACCs were annotated as fragments of *Acidovorax*, *Acinetobacter*, *Aeromonas*, *Methylobacterium*, *Methyloversatilis*, *Mycobacterium*, *Polaromonas*, or *Pseudomonas*, except for those unclassified. Among them, 34.5% of the ACCs were identified as sequence fragments of *Pseudomonas*, frequently carrying multidrug-related ARGs with a percentage of 80% (Fig. 5). The multidrug resistance genes *m* *ex*F and hydrophobe_amphiphile efflux-1 (HAE1) family protein were frequently carried by *Pseudomonas*. Similarly to a previous study, 82% of the *Pseudomonas aeruginosa* strains isolated from a hospital wastewater treatment plant were resistant to multiple antimicrobial drugs [39]. *P. aeruginosa* is a notoriously difficult-to-treat pathogen that can cause severe disease and infections. In *P. aeruginosa*, the efflux mechanism for antibiotic resistance may pose a great challenge to antibiotic development [40]. Thus, the high frequency of ARGs carried by *Pseudomonas* in drinking water may increase the risk of infection and antibiotic ineffectiveness in human beings. Additionally, all the *Methyloversatilis* and *Polaromonas* contigs were observed to carry multidrug- and bacitracin-related ARGs, respectively. Moreover, two contigs were able to be assigned to species level. The contig of *P. aeruginosa*, S13_contig_64868 carried class A beta-lactamase resistance gene, and S08_contig_54033 annotated as contig of *Hylemonella gracilis* carried bacitracin undecaprenol kinase. The taxonomic annotation of ACCs greatly strengthens the identification of possible ARG hosts in drinking water samples.

### The spatial distribution of bacterial community

Totally, 26,862 bacterial OTUs were observed in at least one of these tap water samples. Among them, the highest bacterial diversity across all samples, 5018 OTUs, was observed in Tibet tap water of mainland China, followed by 4302 OTUs in water from Xinjiang, and 4262 OTUs in water from Inner Mongolia in northwestern China. The lowest bacterial diversity was observed in Macau (122 OTUs), followed by California of the USA (210 OTUs), and Sichuan province of China (226 OTUs). Based on the OTUs and corresponding abundances, PCoA was performed to compare the spatial variations of bacterial community using weighted Unifrac distance, which considered both species abundance and phylogeny (Additional file 1 Figure S6). Overall, differences in phylogenetic diversity and abundance of OTUs were obvious across drinking water samples. Notably, the composition of bacterial communities was more similar among the triplicated samples collected from the same tap, triplicated DNA extractions from the same sample, DNA extractions using different kits, and triplicated PCRs using primers with different barcodes. Thus, in the present study, DNA extraction strategy and PCR amplifications using primers with different barcodes are not expected to be factors influencing the detection of microbial compositions in tap water samples.

**Fig. 6:**
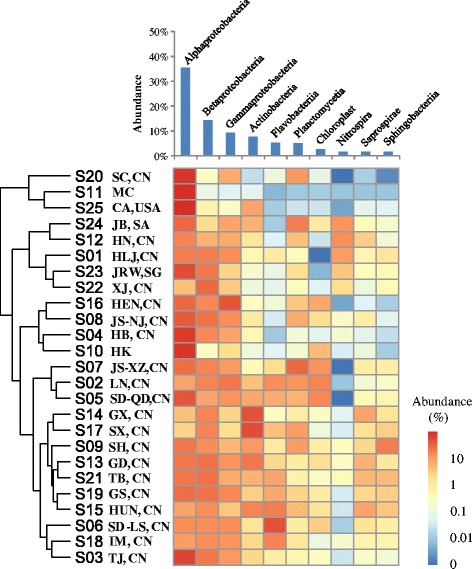
The top 10 bacterial classes in drinking water samples and their cluster patterns. Bar chart shows the average abundance of these top 10 bacterial classes in samples

The histogram in Fig. 6 shows the percentages of the top 10 bacterial classes (accounting for a total abundance of 84.8%) that were most abundant within tap water. The most abundant bacterial classes were *Alphaproteobacteria*, followed by *Betaproteobacteria*, *Gammaproteobacteria*, and *Actinobacteria.* Significantly, the cluster patterns revealed by weighted UniFrac tree (Fig. 6) showed that drinking water sample collected from Macau (S11) closely resembles the sample from California in the USA (S25) based on bacterial community analysis. The Macau and California samples had low OTU diversities of 122 and 210 and had significantly high percentages of *Alphaproteobacteria* at 99.6 and 90.2%, respectively. As shown in Fig. 3, ten OTUs were widely spread with a detection frequency of 80% (20 out of 25 samples), accounting for 16.8% of the total abundance. To further explore the shared, specialist, and generalist bacteria in tap water samples, all of the OTUs that could be annotated at genus level by SSU SILVA database were summarized to obtain the matrix of percentages at genus level in all tap water samples. About 42% of 16S rRNA gene sequences were annotated at genus level (686 genera). Nine genera, *Sphingomonas*, *Pseudomonas*, *Mycobacterium*, *Acinetobacter*, *Hyphomicrobium*, *Planctomyces*, *Sediminibacterium*, *Legionella*, and *Rhodobacter* were present in all tap water samples, and the average abundance was 0.7–14.9%. There were 36 generalist genera (occurrence observed in at least 80% of samples) found to be widely present in tap water samples, accounting for total percentage as 84.9%. The most dominant genus, *Sphingomonas* spp., is known to be aerobic and able to form biofilms. *Sphingomonas* spp. are often investigated in oligotrophic environments [41] and reported to be present in drinking water distribution systems [10, 42, 43]. However, none of the ACCs identified in the present study were annotated as *Sphingomonas* (Fig. 5 **)**, indicating their low frequency of carrying ARGs in drinking water. The dominant genera, *Pseudomonas* (10.0%), *Mycobacterium* (3.4%) and *Acinetobacter* (3.3%), were found to carry aminoglycoside, bacitracin, multidrug, and sulfonamide resistance genes from metagenomic analysis of drinking water samples **(**Fig. 5 **)**. Some *Pseudomonas* spp. (e.g., *P. aeruginosa*), *Mycobacterium* spp. (e.g., *M. avium*), and *Acinetobacter* spp. (e.g., *A. baumannii*) have been considered as opportunistic pathogens, and their capacity of thriving in drinking water supply systems could increase the risks of the exposure and spread of ARGs in drinking water. Thus, the dominance of these ARG-carrying bacteria observed in drinking water at the user end should receive more attention, and their potential negative effects merit further study.

### The horizontal gene transfer potential for ARGs among bacterial population

**Fig. 7:**
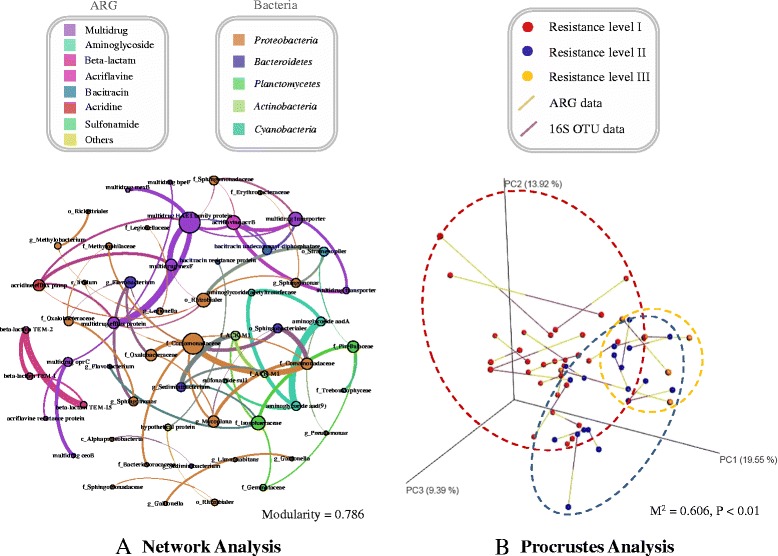
Correlation analysis of ARGs and bacterial community based on network and Procrustes analysis. **a**. Correlation-based network statistical analysis reveals the co-occurrence patterns among ARG subtypes and bacterial OTUs. The nodes were colored according to modularity class. The size of each node is proportional to its number of connections. The edges present the correlation between two nodes. A connection represents a strong (Spearman’s rank correlation coefficient *ρ* > 0.6) and significant (*P* value < 0.01) correlation. Modularity index 0.786. **b**. Procrustes analysis depicts correlation between ARG content (Bray-Curtis) and bacterial community (Bray-Curtis), *M* ^2^ = 0.606, *P* < 0.01

Previous metagenomic analysis-based study revealed the exchange of ARGs between clinical pathogens and environmental bacteria [44], illustrating that ARGs could be transferred between different environments via specific bacteria, especially pathogens. Another study explored the potential HGT frequency among bacterial populations by using Procrustes analysis, and a low HGT frequency of resistomes (*M* ^2^ ≤ 0.5) was observed among soil bacteria [12]. Because drinking water exerts direct exposure to human beings, there is a pressing demand to determine the likelihood of the transmission of ARGs to specific bacteria and the probable hosts of ARGs. Here, network analysis together with Procrustes analysis was applied to explore the correlation between drinking water resistomes and bacterial population (Fig. 7). The modularity index of 0.786 indicated that the formed ARG-bacteria network had a modular structure [45], while positive correlations were frequently found in ARG–ARG and bacteria–bacteria pairings. No significant correlation was observed between ARGs and bacterial population using network analysis. To further validate the absence of significant correlation between ARGs and bacterial population, Procrustes analysis was used based on a one-way ANOVA test with Tukey post-hoc tests [12]. Similarly, the result of Procrustes analysis (*M* ^2^ = 0.606, *P* < 0.01) showed that the ARG content did not correlate with bacterial community, different from the strong correlation observed in soil samples, suggesting the potential of HGT of ARGs in drinking water microbiota [12]. The possible HGT of ARGs indicated by the correlation-based Procrustes analysis in drinking water microbiota may enhance the risks to human health. Thus, further observation and additional analyses are required to study the putative HGT of ARGs in drinking water systems.

## Conclusions

In this study, a catalog of antibiotic resistome in drinking water was established and the host-tracking of ARGs was conducted via a large-scale survey using metagenomic approach. In total, 181 ARG subtypes belonging to 16 ARG types were detected with an abundance range from 2.8 × 10^−2^ to 4.2 × 10^−1^ *capc*. The highest abundance was observed in northern China. The dominant ARGs in the drinking water samples include bacitracin, multidrug, aminoglycoside, sulfonamide, and beta-lactam resistance genes. Moreover, metagenomic assembly based host-tracking revealed that 80% of the ARG-carrying contigs originating from *Pseudomonas* spp. carried multidrug resistance genes. The findings of this study should propel the global surveillance and risk assessment of ARGs in drinking water onto the agendas of water supply authorities. This will aid to prevent both the proliferation of ARGs in drinking water and their horizontal transfer to pathogenic microbes that might cause more cases of antibiotic ineffectiveness and threat to public health.

## Additional file

Additional file 1: S1. Data filtration. **S2.** Illumina MiSeq sequencing for 16S rRNA genes. **S3.** Co-occurrence patterns among ARGs. **Table S1.** Basic information about drinking water samples. **Table S2.** Information about 16S rRNA gene analysis in drinking water samples. **Table S3.** The primers (including barcode, pad, and linker) used for 16S rRNA gene amplification. **Table S4.** Detected ARG types and corresponding abundances in drinking water. **Table S5.** Detected ARG subtypes and corresponding abundances in drinking water. **Table S6.** The resistance level in drinking water samples. **Table S7.** Abundance and frequency of ARG types in drinking water samples. **Table S8.** Co-occurring ARGs subtypes of network modules. **Table S9.** Basic information about 56 environmental samples for comparison of ARGs with drinking water samples. **Table S10.** Occurrence of 9 generalist ARG subtypes that were prevalent in drinking water. **Table S11.** Assembly statistics for drinking water metagenomic sequencing data. **Table S12.** Types and subtypes of ARGs carried by ACCs and their occurrence in drinking water. **Table S13.** Bacterial taxonomy of ARG-carrying contigs in the lowest annotation level and ARGs they carried. **Table S14.** Bacterial OTUs and their abundances in drinking water. **Figure S1.** Collection locations of drinking water samples. **Figure S2.** Cluster analysis of drinking water metagenomes based on abundance of ARG subtypes (Euclidean). **Figure S3.** Abundance of the top 50 ARG subtypes detected in tap water samples. **Figure S4.** Correlation-based network statistical analysis reveals co-occurrence patterns among ARG subtypes. **Figure S5.** Two-dimensional principal coordinate analysis plots show the ARG composition differences among the 81 environmental samples from 8 ecosystems. **Figure S6.** Two-dimensional principal coordinate analysis plots show the microbial community difference among tap water samples. (ZIP 3889 kb)
