# Supplementary material for: Catalogue of antibiotic resistome and host-tracking in drinking water deciphered by a large scale survey
Source: Microbiome. 2017 Nov 28;5:154. doi: 10.1186/s40168-017-0369-0 (PMC5704573; doi:10.1186/s40168-017-0369-0)
Supplement: Additional file 1: S1. — Data filtration. S2. Illumina MiSeq sequencing for 16S rRNA genes. S3. Co-occurrence patterns among ARGs. Table S1. Basic information about drinking water samples. Table S2. Information about 16S rRNA gene analysis in drinking water samples. Table S3. The primers (including barcode, pad, and linker) used for 16S rRNA gene amplification. Table S4. Detected ARG types and corresponding abundances in drinking water. Table S5. Detected ARG subtypes and corresponding abundances in drinking water. Table S6. The resistance level in drinking water samples. Table S7. Abundance and frequency of ARG types in drinking water samples. Table S8. Co-occurring ARGs subtypes of network modules. Table S9. Basic information about 56 environmental samples for comparison of ARGs with drinking water samples. Table S10. Occurrence of 9 generalist ARG subtypes that were prevalent in drinking water. Table S11. Assembly statistics for drinking water metagenomic sequencing data. Table S12. Types and subtypes of ARGs carried by ACCs and their occurrence in drinking water. Table S13. Bacterial taxonomy of ARG-carrying contigs in the lowest annotation level and ARGs they carried. Table S14. Bacterial OTUs and their abundances in drinking water. Figure S1. Collection locations of drinking water samples. Figure S2. Cluster analysis of drinking water metagenomes based on abundance of ARG subtypes (Euclidean). Figure S3. Abundance of the top 50 ARG subtypes detected in tap water samples. Figure S4. Correlation-based network statistical analysis reveals co-occurrence patterns among ARG subtypes. Figure S5. Two-dimensional principal coordinate analysis plots show the ARG composition differences among the 81 environmental samples from 8 ecosystems. Figure S6. Two-dimensional principal coordinate analysis plots show the microbial community difference among tap water samples. (ZIP 3889 kb) [file 40168_2017_369_MOESM1_ESM.zip › Additional file 1_20171017.pdf]

Supporting Information

For

Catalogue of Antibiotic Resistome and Host-tracking in Drinking Water Deciphered by a Large Scale Survey

Liping Ma[1£], Bing Li[2£], Xiao-Tao Jiang[1], Yu-Lin Wang[1], Yu Xia[1], An-Dong Li[1] and Tong Zhang[1*]

[1]Environmental Biotechnology Laboratory, The University of Hong Kong, Hong Kong

[2]Graduate School at Shenzhen, Tsinghua University, China

*Correspondence, zhangt@hku.hk

[£]These authors contributed equally to this work.

Total pages: 32

Table: 14

Figure: 6

Legends

S1 Data filtration………………………………………………….…………….. (page 4)

S2 Illumina MiSeq sequencing for 16S rRNA genes…….…………………….... (page 4)

S3 Co-occurrence patterns among ARGs……………………..……………......(page 5)

Table S1 Basic information about drinking water samples…..……………….(page 7)

Table S2 Information about 16S rRNA gene analysis in drinking water samples……………………………………………………….…….…….(page 9)

Table S3 The primers (including barcode, pad and linker) used for 16S rRNA gene amplification…………………………………………………………….(page 12)

Table S4 Detected ARG types and corresponding abundances in drinking water.

Table S5 Detected ARG subtypes and corresponding abundances in drinking water.

Table S6 The resistance level in drinking water samples…………………….(page 14)

Table S7 Abundance and frequency of ARG types in drinking water samples……………………………………………………………..…(page 15)

Table S8 Co-occurring ARGs subtypes of network modules.…………...…. (page 16)

Table S9 Basic information about 56 environmental samples for ARGs comparison with drinking water samples………………………………..…….……..(page 18)

Table S10 Occurrence of 9 generalist ARG subtypes that were prevalent in drinking water……………………………………………………….……..…...(page 22)

Table S11 Assembly statistics for drinking water metagenomic sequencing data…..(page 23)

Table S12 Types and subtypes of ARGs carried by ACCs and their occurrence in drinking water…………………………………………………….……..(page 24)

Table S13 Bacterial taxonomy of ARG-carrying contigs in the lowest annotation level and ARGs they carried.

Table S14 Bacterial OTUs and their abundances in drinking water.

Fig. S1 Collection locations of drinking water samples…………..………(page 26)

Fig. S2 Cluster analysis of drinking water metagenomes based on abundance of ARG subtypes (Euclidean)……………… ………………………….…….……. (page 27)

Fig. S3 Abundance of the top 50 ARG subtypes detected in drinking water samples.………..…………………………………………………………...(page 28)

Fig. S4 Correlation-based network statistical analysis reveals co-occurrence patterns among ARG subtypes.…………………...…………………………….……..(page 29)

Fig. S5 Two-dimensional principal coordinate analysis plots show the ARG composition differences among the 81 environmental samples from 8 ecosystems (Bray-Curtis) ……………………...………………..…………………...…....(page 30)

Fig. S6 Two-dimensional principal coordinate analysis plots show the microbial community difference among tap water samples…………….…………………….(page 31)

S1 Data filtration

To guarantee the quality of the down-stream analysis, data filtration of generated raw metagenomic data of 25 tap water samples was performed to remove the raw reads containing three or more ambiguous nucleotides, or with an average quality score below 20, or with a length less than 100 nt and artificially identical reads [1].

S2 Illumina MiSeq sequencing for 16S rRNA genes

The V4 region (~265 nucleotides) of the 16S rRNA gene sequences was amplified using F515 (5′-GTGCCAGCMGCCGCGGTAA-3′) and R806 (5′-GGACTACHVGGGTWTCTAAT-3′) from genomic DNA of microorganisms of tap water samples. Dual-index sequencing strategy for primers (adapter + barcode + pad + linker + primer, **Table S3**) and reaction conditions applied in this study followed a previous study [2]. Three PCR assays were performed to avoid the variations, and purified triplicate PCR amplicons were pooled together and then performed on Illumina MiSeq PE250 (BGI). Drinking water samples were amplified for the V4 regions of 16S rRNA gene of microorganisms, with the sequence number of 32,525 ~ 147,248. Additionally, three blank samples not filtered with tap water were used for control. Two samples were selected for amplification using primers with two additional paired barcodes to evaluate the biases of using primers with different barcodes. Besides, one sample was selected using different DNA extraction kits to assess the impacts of kits on DNA extractions (Kit-1: FastDNA SPIN Kit for Soil (MP Biomedicals, France); Kit-2: FastDNA SPIN Kit (MP Biomedicals, France); Kit-3: PowerWater DNA Isolation Kit (Mo Bio, United States); Kit-4: UltraClean Microbial DNA Isolation Kit (Mo Bio, United States)). All 16S rRNA gene sequences generated from tap water samples were deposited into NCBI SRA under the BioProject of PRJNA305188.

The generated 16S rRNA gene sequences were analyzed using Mothur software package by following the analysis protocol of MiSeq SOP [2]. The samples were separated according to barcodes using a custom-made Perl script, and then submitted onto Mothur. Briefly, sequence pairs were assembled into contigs, and any contig with ambiguous base and no longer than 275 bp was removed. The filtered contig sequences were aligned to SILVA database (SSU_Ref database version 123) to conduct error removals. Then chimera contigs were identified and removed using UCHIME [3]. After filtration, the operational taxonomic unit (OTU) table was constructed from clean sequences using QIIME (version 1.9.1) [4] including picking up OTUs, picking and aligning representative sequences, taxonomic assignment, and building up a phylogenetic tree of OTUs. Finally, the number of 16S rRNA genes of each sample was normalized to 30,000 sequences, and then the obtained matrix table of OTUs and their abundances in tap water samples was ready for downstream network analysis.

S3 Co-occurrence patterns among ARGs

The co-occurrence patterns among ARG subtypes were explored using network inference based on strong ($\rho > 0.6$) and significant (P-value < 0.01) correlations [5] in occurrence within at least 40% tap water samples. **Fig. S3** consists of 37 nodes (ARG subtypes) and 67 edges (connections). The modularity index of 0.493 indicated that the formed ARG-ARG network had a modular structure [6]. It is shown in **Fig. S3** and **Table S8** that the ARG-ARG network is parsed into 10 major modules based on the modularity class, that is, correlations. 18 of 37 total nodes belonged to the three largest modules: Modules I, II and III. The most densely connected node of each module is defined as ‘hub’, considered as the occurrence indicator of other nodes (ARG subtypes) belonging to that module in the tap water environment [7]. Significantly, multidrug HAE1 family protein is the hub of Module I, acting as the ARG indicator to estimate the quantity of associated ARGs in Module I, including *mex*B, *mex*G, multidrug RND (Resistance-nodulation-division) protein, acriflavine *acr*B, multidrug transportor and bacitracin undecaprenyl diphosphatase. They are likely to be carried by common specific bacteria, shared by different tap water samples [7].

Additionally, the co-occurrences of ARGs were frequently observed among ARGs belonging to the same ARG types, for example: beta-lactam (TEM-1, TEM-2 and TEM-15 in Moduel VI), multidrug (*bpe*F, *opr*C and *ceo*B in Model IV; *mex*E, *mex*F and *opr*N in Model I) and aminoglycoside (*aad*A, *aad*B, *aad*(9) in Module II). Interestingly, some of the ARG associations revealed by this ARG-ARG network have been proposed by previous studies as ARG-combinations harbored in specific microbial taxa. For example, the gene combination of multidrug *mex*E-*mex*F-*opr*N has been detected as the positive regulated multidrug efflux system of *Pseudomonas aeruginosa* [8]. It was also observed that sulfonamide-ARG *sul*1 and aminoglycoside-ARGs *aad*A and *aad*B co-occurred on class 1 integron of *Salmonella enterica* [9]. These previous findings well supported the ARG combinations revealed by network analysis in the present study.

Table S1 Basic information about collected drinking water samples.

Sample ID	Sampling location (city, country)	Abbreviation of sampling location	Latitude and Longitude	Sampling time (mm-year)	Person responsible for sample collection
S01	Fujin, Heilongjiang, China	HLJ, CN	132:03E, 47:25N	01-2015	Dr. Yuanyuan Wei
S02	Dalian, Liaoning, China	LN, CN	121:38E 38:54N	02-2015	Dr. Yu Xia
S03	Tianjin, China	TJ, CN	117:10E 39:10N	01-2015	Dr. Yuchen Pang
S04	Botou, Hebei, China	HB, CN	116:20E 38:03N	02-2015	Dr. Wenjun Sun
S05	Qingdao, Shandong, China	SD-Q, CN	120:19E 36:04N	02-2015	Dr. Bing Li
S06	Linshu, Shandong, China	SD-L, CN	118:43E 34:52N	02-2015	Mr. Yulin Wang
S07	Xuzhou, Jiangsu, China	JS-X, CN	117:11E 34:15N	02-2015	Ms. Anni Zhang
S08	Nanjing, Jiangsu, China	JS-N, CN	118:46E 32:03N	02-2015	Dr. Andong Li
S09	Shanghai, China	SH, CN	121:26E 31:12N	02-2015	Dr. Liping Ma
S10	Hong Kong, China	HK	114:15E 22:15N	03-2015	Dr. Liping Ma
S11	Macau, China	MC	113:32E 22:12N	02-2015	Mr. Xiaotao Jiang
S12	Haikou, Hainan, China	HN, CN	120:48E 40:36N	03-2015	Dr. Ke Yu
S13	Guangzhou, Guangdong, China	GD, CN	113:18E 23:10N	02-2015	Dr. Ying Yang

S14	Nanning, Guangxi, China	GX, CN	108:21E 22:47N	02-2015	Dr. Yanping Mao
S15	Wugang, Hunan, China	HUN, CN	111:53E 29:17N	01-2015	Ms. Yu Deng
S16	Zhengzhou, Henan, China	HEN, CN	113:42E 34:44N	01-2015	Dr. Yuanqing Chao
S17	Jinzhong, Shanxi, China	SX, CN	112:45E 37:42N	02-2015	Ms. Yu Deng
S18	Alashan, Inner Mongolia, China	IM, CN	105:08E 39.36N	02-2015	Dr. Bing Li
S19	Yinchuan, Gansu, China	GS, CN	106:13E 38:28N	03-2015	Dr. Liping Ma
S20	Chengdu, Sichuan, China	SC, CN	104:04E 30:39N	02-2015	Prof. Tong Zhang
S21	Xigaze, Tibet, China	TB, CN	88:49E 29:16N	02-2015	Dr. Liping Ma
S22	Akesu, Xinjiang, China	XJ, CN	80:18E 41:09N	02-2015	Ms. Yu Deng
S23	Jurong West, Singapore	JRW, SG	103:43E 1:21N	12-2014	Dr. Liping Ma
S24	Johannesburg, South Africa	JB, SA	28:00E 26:10S	01-2013	Dr. Bing Li
S25	Stanford, California, United States	CA, USA	122:10W 37:25N	12-2012	Dr. Bing Li

Table S2 Information about 16S rRNA gene analysis in drinking water samples.

Primer[a]	Sample ID	Sampling points	Number of obtained 16S rRNA gene sequences	Diversity of OTUs	Remarks
F1R3	S24	Johannesburg, Africa	32525	1232	
F1R7	S14	Nanning, Guangxi province, China	57742	1810	
F1R10	S17	Jinzhong, Shanxi province, China	46832	1120	
F2R1	S06	Jiaozhou, Qingdao, Shandong, China	68081	860	
F2R3	S07	Xuzhou, Jiangsu province, China	51810	393	
F2R4	S20	Chengdu, Sichuan province, China	62986	226	
F2R6	S03	Tianjin, China	77767	524	
F3R1	S15	Wugang, Hunan province, China	74051	1060	
F3R5	S08	Nanjing, Jiangsu province, China	114927	1226	
F3R11	S18	Alashan league, Inner Mongolia, China	102732	4262	
F3R12	S16	Zhengzhou, Henan province, China	114374	411	
F4R5	S09	Shanghai, China	57981	1398	
F5R2	S05	Linyi, Shandong province, China	121638	1012	
F5R5	S22	Aksu, Xinjiang province, China	65494	4302	

F5R9	TW57	BlankA	169		
F5R10	TW58	BlankB	177	NA	Control
F5R11	TW59	BlankC	279		
F6R6	S04	Cangzhou, Hebei province, China	80691	621	
F6R9	S02	Dalian, Liaoning province, China	116940	672	
F7R3	S21	Xigaze, Tibet province, China	103499	5018	
F8R7	S23	Singapore	90050	1135	
F8R9	S01	Fujin, Heilongjiang province, China	75369	3005	
F8R12	S11	Macau, China	125431	122	
F9R5	S25	Stanford, California, USA	91815	210	
F9R7	S19	Yinchuan, Gansu province, China	111860	1280	
F9R12	S13	Guangzhou, Guangdong province, China	147248	1021	
F10R9	S10	Hong Kong Island, Hong Kong, China-D1	131539	275	
F10R10	TW118	Hong Kong Island, Hong Kong, China-D2	428	NA	
F10R11	TW119	Hong Kong Island, Hong Kong, China-D3	256996	329	
F11R4	S12	Haikou, Hainan province, China	139007	2270	

F11R7	TW127	Hong Kong Island, Hong Kong, China-DNA1	239	NA	Triplicate DNA extraction for the same sample
F11R8	TW128	Hong Kong Island, Hong Kong, China-DNA2	156723	223	
F11R9	TW129	Hong Kong Island, Hong Kong, China-DNA3	228916	254	
F11R10	TW130	Hong Kong Island, Hong Kong, China-Kit-1	132730	350	Kit-1
F11R11	TW131	Hong Kong Island, Hong Kong, China-Kit-2	183952	354	Kit-2
F11R12	TW132	Hong Kong Island, Hong Kong, China-Kit-3	184058	317	Kit-3
F12R1	TW133	Hong Kong Island, Hong Kong, China-D-Kit-4	140	NA	Kit-4
F12R2	TW134	Triplicate(2)_Xigaze, Tibet province, China	137886	4850	
F12R3	TW135	Triplicate(3)_Xigaze, Tibet province, China	137433	4996	
F12R4	TW136	Triplicate(2)_Fujin, Heilongjiang province, China	110167	3105	
F12R5	TW137	Triplicate(3)_Fujin, Heilongjiang province, China	187432	2960	

Kit-1: FastDNA SPIN Kit for Soil (MP Biomedicals, France)

Kit-2: FastDNA SPIN Kit (MP Biomedicals, France)

Kit-3: PowerWater DNA Isolation Kit (Mo Bio, United States)

Kit-4: UltraClean Microbial DNA Isolation Kit (Mo Bio, United States)

[a] The information of the primers used in this study were summarized in **Table S3**.

Drinking water samples (TW57~59, TW118~119, TW127~TW137) were for control and testing for the biases of DNA extraction, biological triplicates and PCR reactions.

Table S3 The primers (including barcode, pad and linker) used for 16S rRNA gene amplification.

ID	Forward
V4F1	AATGATACGGCGACCACCGAGATCTACACATCGTACGTATGGTAATTGTGTGCCAGCMGCCGCGGTAA
V4F2	AATGATACGGCGACCACCGAGATCTACACACTATCTGTATGGTAATTGTGTGCCAGCMGCCGCGGTAA
V4F3	AATGATACGGCGACCACCGAGATCTACACTAGCGAGTTATGGTAATTGTGTGCCAGCMGCCGCGGTAA
V4F4	AATGATACGGCGACCACCGAGATCTACACCTGCGTGTTATGGTAATTGTGTGCCAGCMGCCGCGGTAA
V4F5	AATGATACGGCGACCACCGAGATCTACACTCATCGAGTATGGTAATTGTGTGCCAGCMGCCGCGGTAA
V4F6	AATGATACGGCGACCACCGAGATCTACACCGTGAGTGTATGGTAATTGTGTGCCAGCMGCCGCGGTAA
V4F7	AATGATACGGCGACCACCGAGATCTACACGGATATCTTATGGTAATTGTGTGCCAGCMGCCGCGGTAA
V4F8	AATGATACGGCGACCACCGAGATCTACACGACACCGTTATGGTAATTGTGTGCCAGCMGCCGCGGTAA
V4F9	AATGATACGGCGACCACCGAGATCTACACCTACTATATATGGTAATTGTGTGCCAGCMGCCGCGGTAA
V4F10	AATGATACGGCGACCACCGAGATCTACACCGTTACTATATGGTAATTGTGTGCCAGCMGCCGCGGTAA
V4F11	AATGATACGGCGACCACCGAGATCTACACAGAGTCACTATGGTAATTGTGTGCCAGCMGCCGCGGTAA
V4F12	AATGATACGGCGACCACCGAGATCTACACTACGAGACTATGGTAATTGTGTGCCAGCMGCCGCGGTAA
**ID**	**Reverse**
V4R1	CAAGCAGAAGACGGCATACGAGATAACTCTCGAGTCAGTCAGCCGGACTACHVGGGTWTCTAAT
V4R2	CAAGCAGAAGACGGCATACGAGATACTATGTCAGTCAGTCAGCCGGACTACHVGGGTWTCTAAT
V4R3	CAAGCAGAAGACGGCATACGAGATAGTAGCGTAGTCAGTCAGCCGGACTACHVGGGTWTCTAAT
V4R4	CAAGCAGAAGACGGCATACGAGATCAGTGAGTAGTCAGTCAGCCGGACTACHVGGGTWTCTAAT

V4R5	CAAGCAGAAGACGGCATACGAGATCGTACTCAAGTCAGTCAGCCGGACTACHVGGGTWTCTAAT
V4R6	CAAGCAGAAGACGGCATACGAGATCTACGCAGAGTCAGTCAGCCGGACTACHVGGGTWTCTAAT
V4R7	CAAGCAGAAGACGGCATACGAGATGGAGACTAAGTCAGTCAGCCGGACTACHVGGGTWTCTAAT
V4R8	CAAGCAGAAGACGGCATACGAGATGTCGCTCGAGTCAGTCAGCCGGACTACHVGGGTWTCTAAT
V4R9	CAAGCAGAAGACGGCATACGAGATGTCGTAGTAGTCAGTCAGCCGGACTACHVGGGTWTCTAAT
V4R10	CAAGCAGAAGACGGCATACGAGATTAGCAGACAGTCAGTCAGCCGGACTACHVGGGTWTCTAAT
V4R11	CAAGCAGAAGACGGCATACGAGATTCATAGACAGTCAGTCAGCCGGACTACHVGGGTWTCTAAT
V4R12	CAAGCAGAAGACGGCATACGAGATTCGCTATAAGTCAGTCAGCCGGACTACHVGGGTWTCTAAT

Table S6 The resistance level in drinking water samples.

	Number of samples	Percentage of samples
Resistance level I (<0.1)	14	56%
Resistance level II (0.1~0.2)	9	36%
Resistance level II (>0.2)	2	4%

Table S7 Abundance and frequency of ARG types in drinking water samples.

ARG type	Average abundance (*capc*)	Frequency in 25 drinking water samples	
Bacitracin	5.05E-02	25	100%
Multidrug	3.52E-02	25	100%
Aminoglycoside	7.85E-03	23	92%
Sulfonamide	7.59E-03	23	92%
Beta-lactam	7.42E-03	25	100%
Others	6.31E-03	25	100%
MLS	3.29E-03	25	100%
Acriflavine	2.49E-03	24	96%
Trimethoprim	8.06E-04	9	36%
Tetracycline	1.29E-03	21	84%
Chloramphenicol	9.01E-04	15	60%
Polymyxin	5.51E-04	12	48%
Acridine	4.89E-04	22	88%
Fosmidomycin	1.18E-04	13	52%
Fosfomycin	8.11E-05	8	32%
Vancomycin	7.52E-05	7	28%

Table S8 Co-occurring ARGs subtypes of network modules.

Module	Number of Node	Co-occurring ARGs subtype	Co-occurring ARGs type
I	6	Multidrug *mex*F	Multidrug
		Multidrug *opr*C	Multidrug
		Multidrug *opr*N	Multidrug
		Multidrug efflux protein	Multidrug
		Multidrug *mex*E	Multidrug
		Acridine efflux pump	Acridine
II	6	Aminoglycoside *aad*A	Aminoglycoside
		Aminoglycoside acetyltransferase	Aminoglycoside
		Aminoglycoside *aad*(9)	Aminoglycoside
		Aminoglycoside *aad*B	Aminoglycoside
		Sulfonamide *sul*1	Sulfonamide
		Unnamed protein	Others
III	6	Multidrug HAE1 family protein	Multidrug
		Multidrug *mex*B	Multidrug
		Multidrug transporter	Multidrug
		Multidrug *mex*G	Multidrug

		Acriflavine *acr*B	Acriflavine
		Bacitracin undecaprenyl-diphosphatase	Bacitracin
IV	5	Multidrug *ceo*B	Multidrug
		Multidrug *opr*C	Multidrug
		Multidrug *bpe*F	Multidrug
		Beta-lactamase	Beta-lactam
		Acriflavine resistance protein	Acriflavine
V	3	Polymyxin *arn*A	Polymyxin
		Multidrug *mex*A	Multidrug
		UDP-glucuronic acid decarboxylase	Others

Table S9 Basic information about 56 environmental samples for ARGs comparison with drinking water samples.

Sample ID	Sample type	Sample sites	Sampling time	Dataset size (Gb)	Sequencing technology	Sequence length (nt)	Data source	Remark
RW01			February, 2012	3				—
RW02			July, 2011	1				—
RW03			July, 2012	3				
RW04			July, 2012	3				
RW05			March, 2013	2.5				—
STP.INF.Summer01			July, August and September 2011	3				—
STP.INF.Winter01			November, December 2011 and January 2012	3				—
STP.INF.Summer02			July and August 2012	4				—
STP.INF.Winter02			December 2011 and January 2012	4				—
STP.EFF.Summer01			July, August, September, 2011	3				—
STP.EFF.Winter01			November, December 2011 and January, 2012	3				—
STP.EFF.Summer02			July and August 2012	4				—
STP.EFF.Winter02			December 2011 and January 2012	4				—

STP.AS01	Activated Sludge		July, 2007	5				—
STP.AS02			January, 2008	5				—
STP.AS03			July, 2008	5				—
STP.AS04			January, 2009	5				—
STP.AS05			July, 2009	5				—
STP.AS06			January, 2010	5				—
STP.AS07			July, 2010	5				—
STP.AS08			January, 2011	5				—
STP.AS09				3				Collected from upper foaming layer
STP.AS10				3				Collected from bulk mixed liquor
STP.AS11				3				
STP.AS12				3				
STP.AS13				3				—

STP.ADS01	Anaerobic digestion sludge	Shatin STP, Hong Kong	September, 2011	1				—
STP.ADS03			March, 2012	3				—
STP.ADS08			April, 2014	3				—
STP.ADS09			April, 2014	3				—
STP.ADS02			September, 2011	1				—
STP.ADS04				3				
STP.ADS05				3				
STP.ADS10			April, 2014	3				—
STP.ADS11			April, 2014	3				—
STP.ADS06		TaiPo STP, Hong Kong	April, 2014	3				—
STP.ADS07		TaiPo STP, Hong Kong	April, 2014	3				—
SD01		Lamma Island, Hong Kong	October, 2011	1				—
SD02		San Tin, Hong Kong	September, 2011	3				—
SD03		Tai Po, Hong Kong	May, 2011	3				—
SD04				3				—
SD05				3				—

SD06				3				—
SD07	Deep sea sediment	Arctic deep sea sediment	October, 2012	3				—
PF1M01				2.5				
PF1M02				2.5				
PF1M03				2.5				
PF8M01				2.5				
PF8M02				2.5				
PF8M03				2.5				
PF.STP.INF	Untreated pig farm wastewater			2.5				—
PF.STP.EFF	Treated pig farm wastewater			2.5				—
CF20D01				2.5				
CF20D02				2.5				
CF80D01				2.5				
CF80D02				2.5				

DWTP: Drinking water treatment plant; STP: Sewage treatment plant

Table S10 Occurrence of 9 generalist ARG subtypes that were prevalent in drinking water.

ARG subtypes that were prevalent in all drinking water	The number of environmental samples that had the ARG subtype						
	**Sediment (n=7)**	**River water (n=5)**	**STP AS (n=13)**	**STP ADS (n=11)**	**STP Effluent (n=4)**	**STP Influent (n=4)**	**Livestock farm samples (n=12)**
Bacitracin__undecaprenol kinase	5	5	13	11	4	4	9
Bacitracin__undecaprenyl-diphosphatase	5	5	13	11	4	4	12
Multidrug__multidrug efflux protein	6	5	13	11	4	4	12
Multidrug__hydrophobe_amphiphile efflux-1 (HAE1) family protein	5	5	13	11	4	4	11
Others__hypothetical protein	7	5	13	11	4	4	12
Beta-lactam__TEM-2	0	0	2	0	1	4	9
Macrolide-lincosamide-streptogramin__macrolide permease protein macB	7	5	13	11	4	4	12
Beta-lactam__TEM-15	0	0	3	1	0	4	8

Table 11 Assembly statistics for drinking water metagenomic sequencing data.

**Drinking water data set**	Sequence number	1.01E+9
	Base number (bp)	1.01E+11
	Assembled contig number	3644369
	Average length of contig (bp)	1112
	Number of ACCs	264
	Average length of ACCs (bp)	6824

Table S12 Types and subtypes of ARGs carried by ACCs and their occurrence frequency in drinking water.

**ARGs carried by ACCs**		**ACCs**		**Drinking water samples**	
ARG type	ARG subtype	Number	Percentage	Number	Percentage
Acriflavine	Acriflavine-acrB	1	0.4%	1	4%
Aminoglycoside	Aminoglycoside-2-adenylytransferase	1	0.4%	1	4%
	Aminoglycoside-2-N-acetyltransferase	4	1.5%	3	12%
	Aminoglycoside-6-N-acetyltransferase	3	1.1%	2	8%
	Aminoglycoside-acetyltransferase	1	0.4%	1	4%
	Aminoglycoside-streptomycin 3-adenylyltansferase_aad(9)	1	0.4%	1	4%
Bacitracin	Bacitracin-bacitracin resistance protein	4	1.5%	3	12%
	Bacitracin-undecaprenol kinase	54	20.5%	20	80%
	Bacitracin-undecaprenyl-diphosphatase	49	18.6%	21	84%
Beta-lactam	Beta-lactam-class A beta-lactamase	1	0.4%	1	4%
	Beta-lactam-class B beta-lactamase	1	0.4%	1	4%
	Beta-lactam-OXA-2	1	0.4%	1	4%
Chloramphenicol	Chloramphenicol-chloramphenicol acetyltransferase	1	0.4%	1	4%
	Chloramphenicol-chloramphenicol and florfenicol resistance protein_exporter	1	0.4%	1	4%
	Chloramphenicol-cmlA9	1	0.4%	1	4%
Erythromycin	Erythromycin-erythromycin esterase	2	0.8%	1	4%
Multidrug	Multidrug-bpeF	8	3.0%	5	20%
	Multidrug-ceoB	2	0.8%	2	8%
	Multidrug-hydrophobe_amphiphile efflux-1 (HAE1) family protein	41	15.5%	14	56%
	Multidrug-mexF	13	4.9%	7	28%
	Multidrug-multidrug efflux protein	42	15.9%	13	52%
	Multidrug-oprN	2	0.8%	2	8%

	Multidrug-sdeY	1	0.4%	1	4%
	Multidrug-transporter	2	0.8%	2	8%
Sulfonamide	Sulfonamide-sul1	9	3.4%	8	32%
	Sulfonamide-sul2	10	3.8%	9	36%
Tetracycline	Tetracycline-TetA(C)	1	0.4%	1	4%
	Tetracycline-TetA(G)	1	0.4%	1	4%
Others	Others-hypothetical protein	3	1.1%	3	12%
	Others-RND protein	1	0.4%	1	4%
	Others-UDP-glucuronic acid decarboxylase_UDP-4-amino-4-deoxy-L-arabinose formyltransferase	1	0.4%	1	4%
	Others-unnamed protein	1	0.4%	1	4%

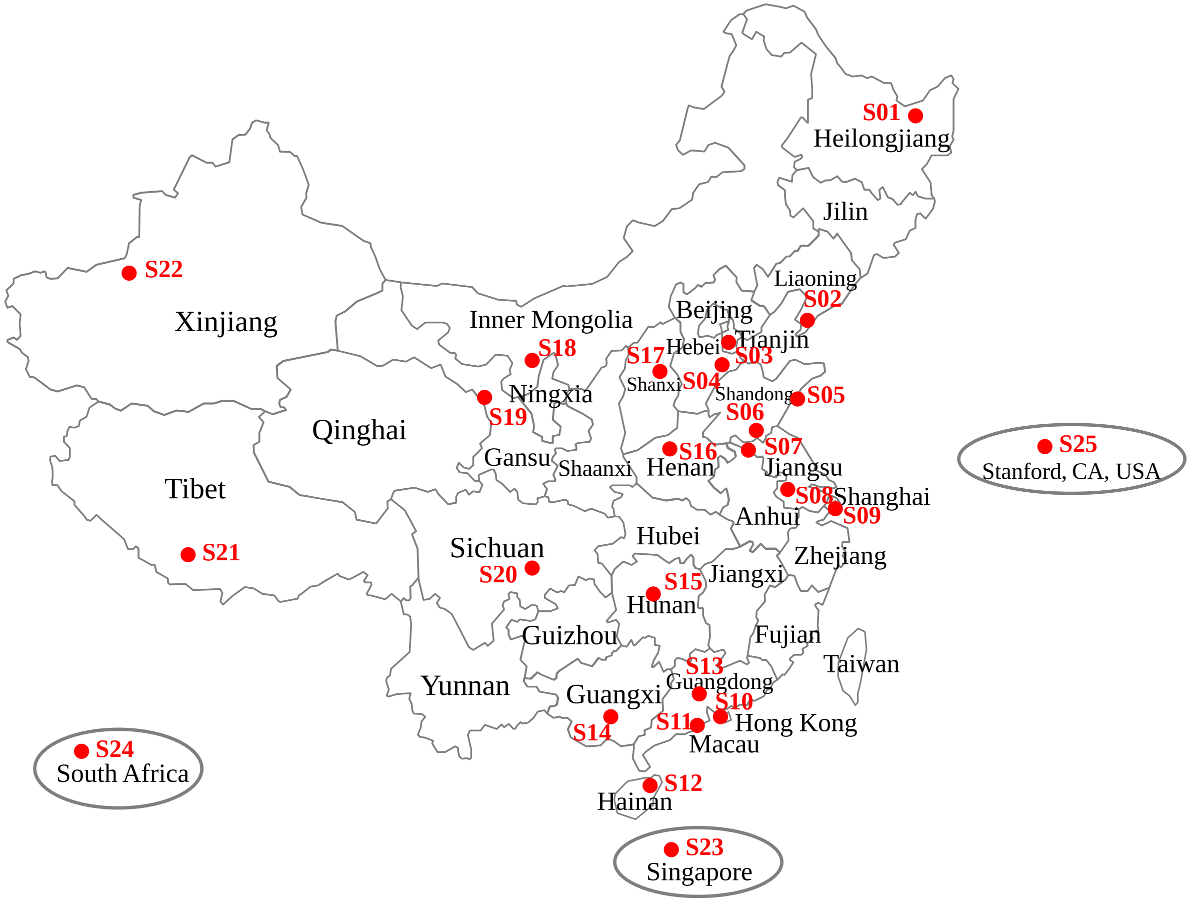

Fig. S1 Collection locations of drinking water samples.

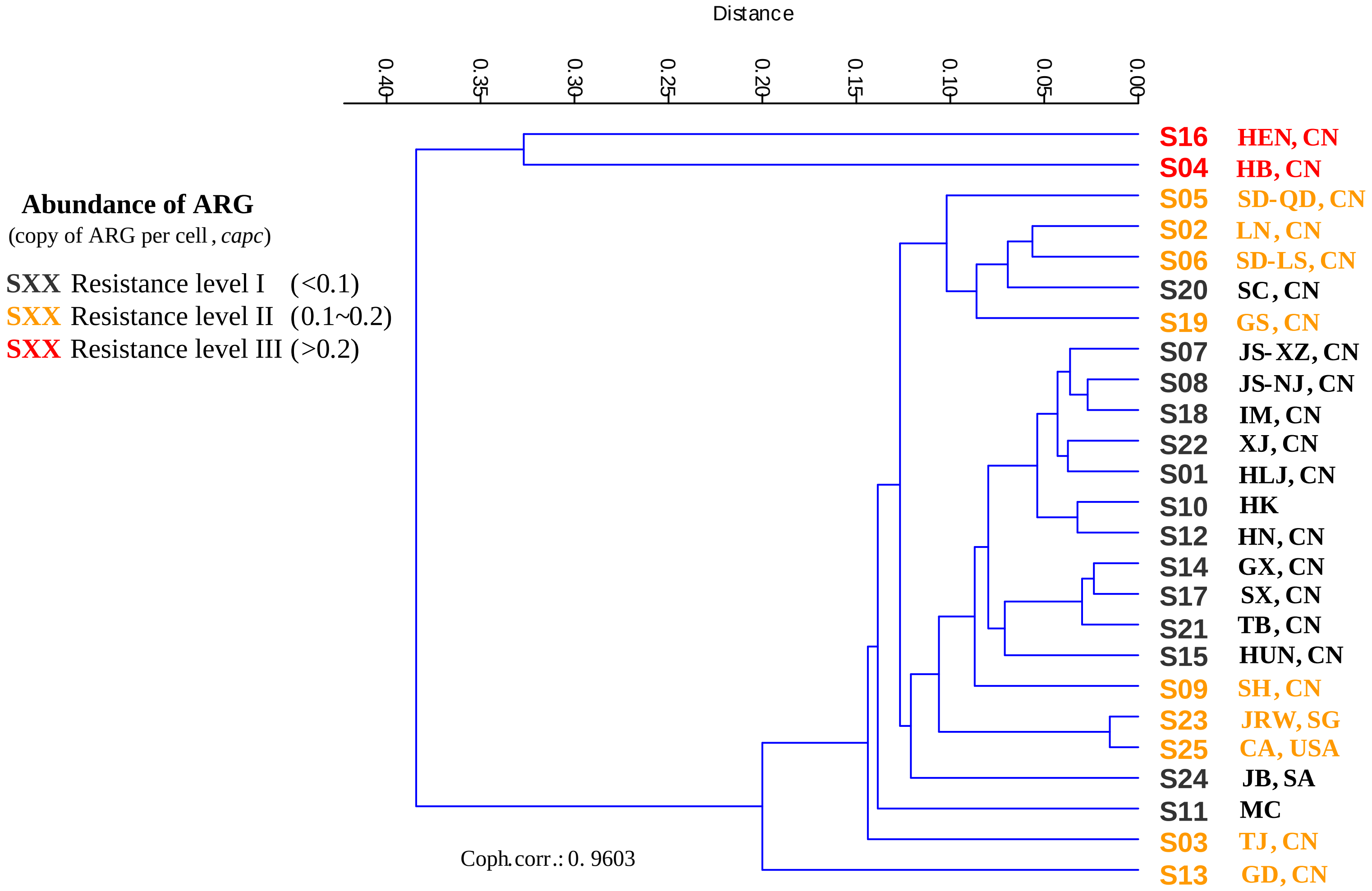

Fig. S2 Cluster analysis of drinking water metagenomes based on abundance of ARG subtypes (Euclidean).

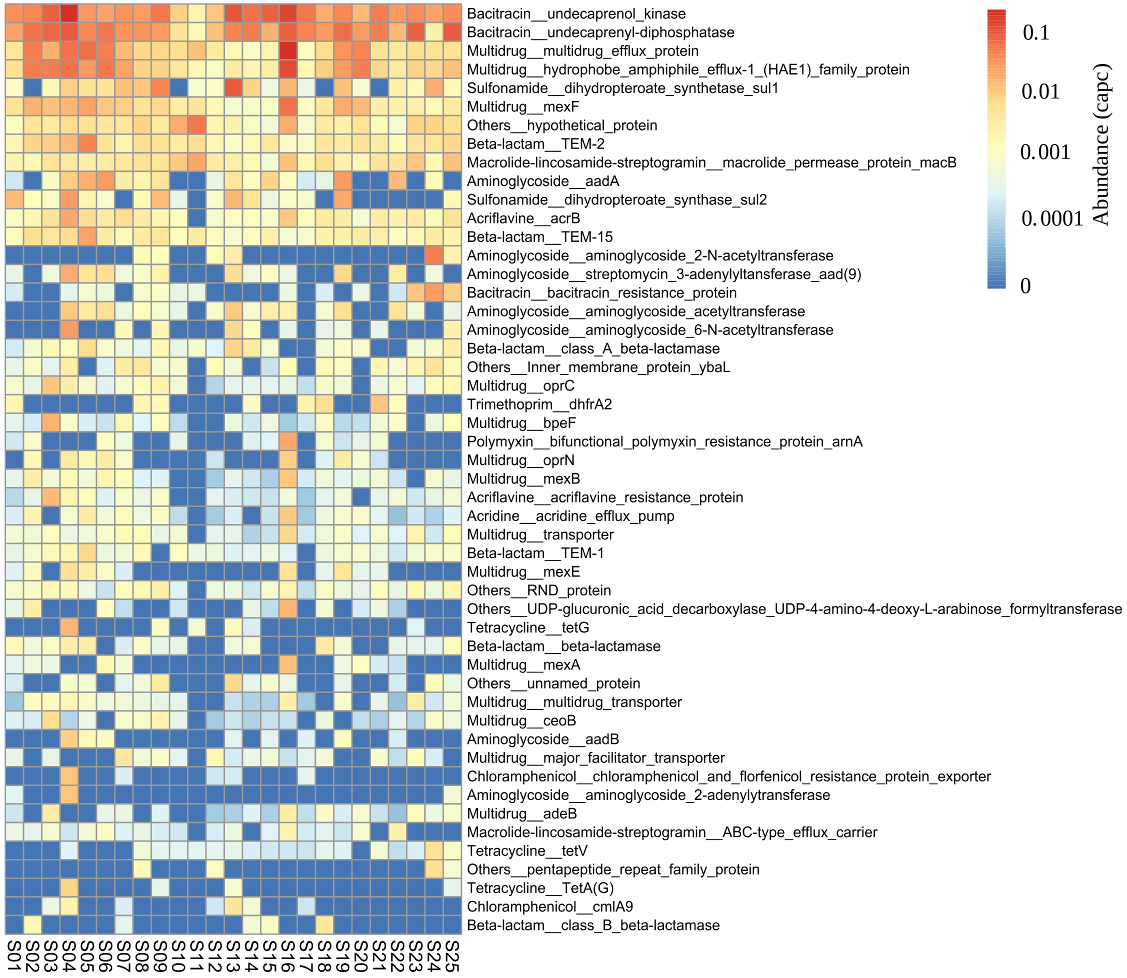

Fig. S3 Abundance of the top 50 ARG subtypes detected in drinking water samples.

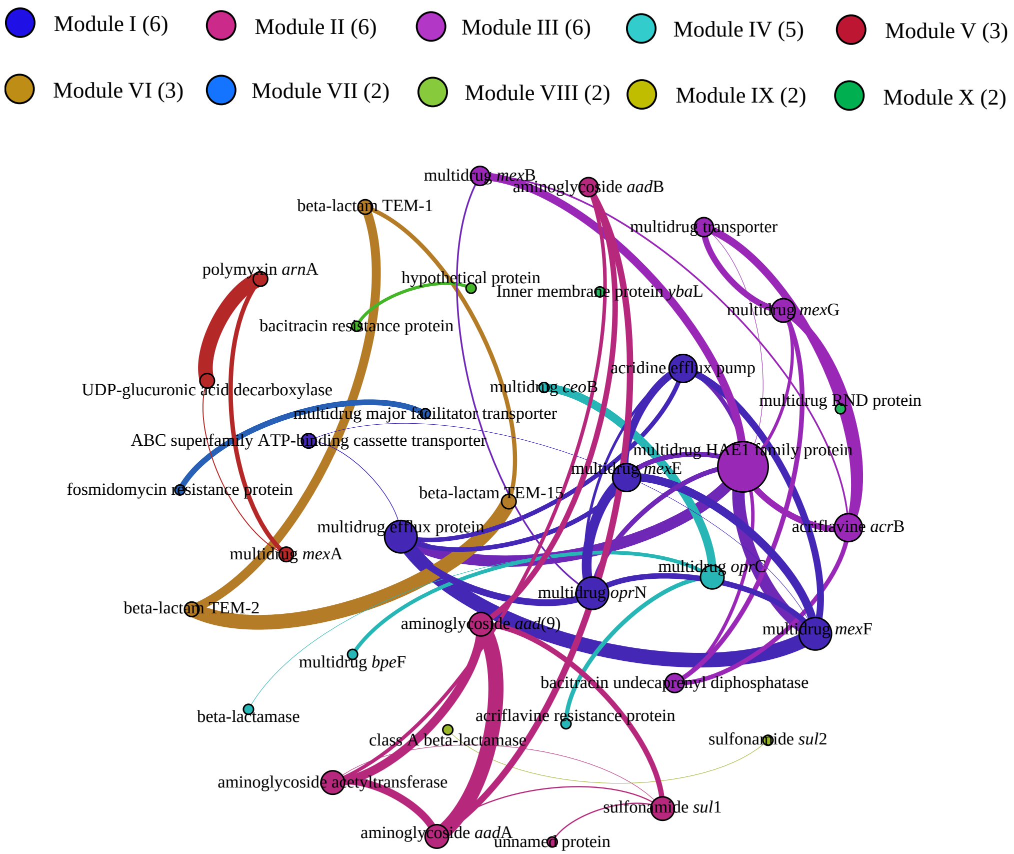

Fig. S4 Correlation-based network statistical analysis reveals co-occurrence patterns among ARG subtypes. The nodes were colored according to modularity class. The size of each node is proportional to its number of connections. The edges present the correlation between two nodes. A connection represents a strong (Spearman's rank correlation coefficient $\rho > 0.6$) and significant (P-value < 0.01) correlation. Modularity index: 0.493.

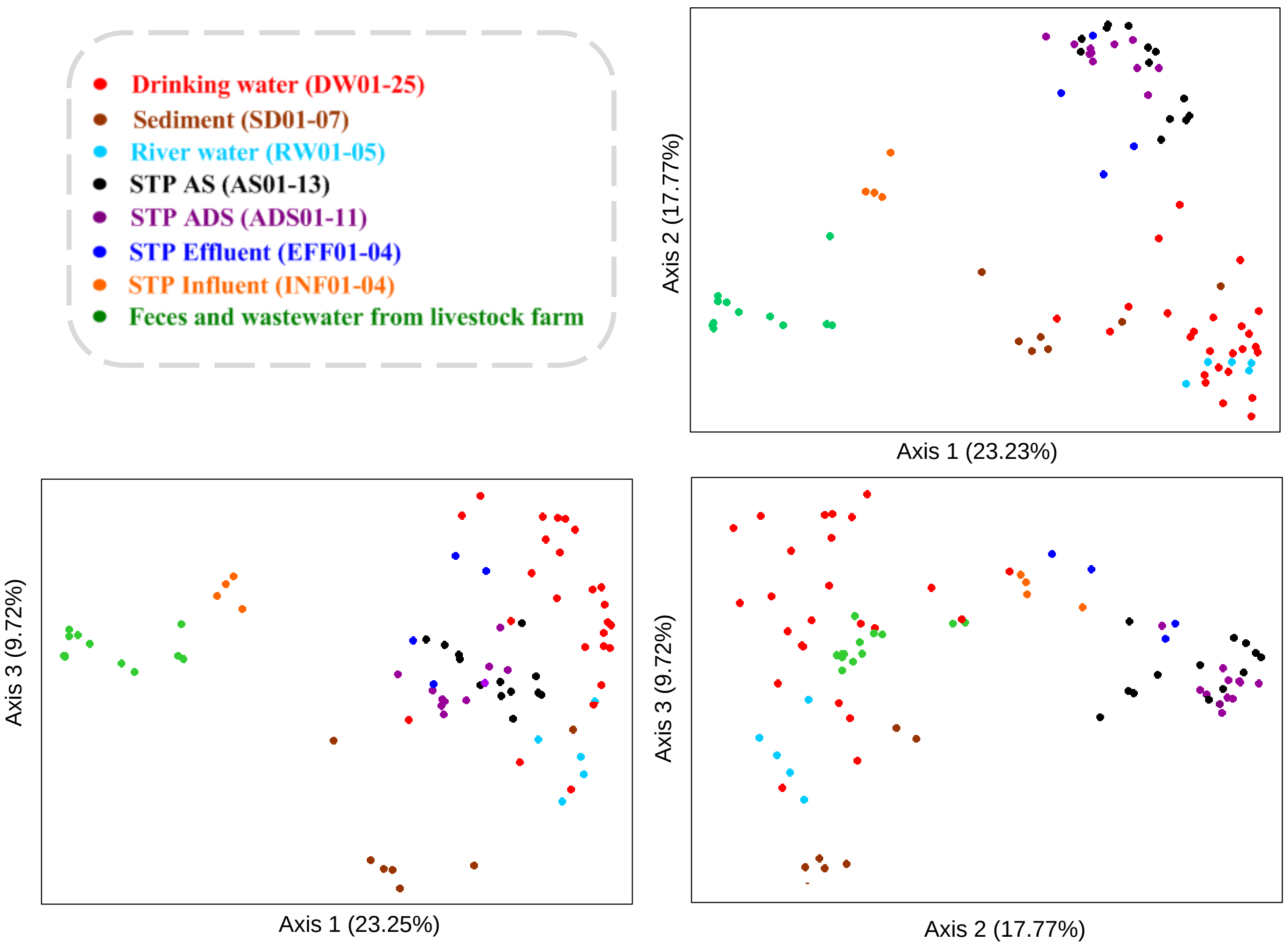

Fig. S5 Two-dimensional Principal Coordinate Analysis plots show the ARG composition differences among the 81 environmental samples from 8 ecosystems (Bray-Curtis).

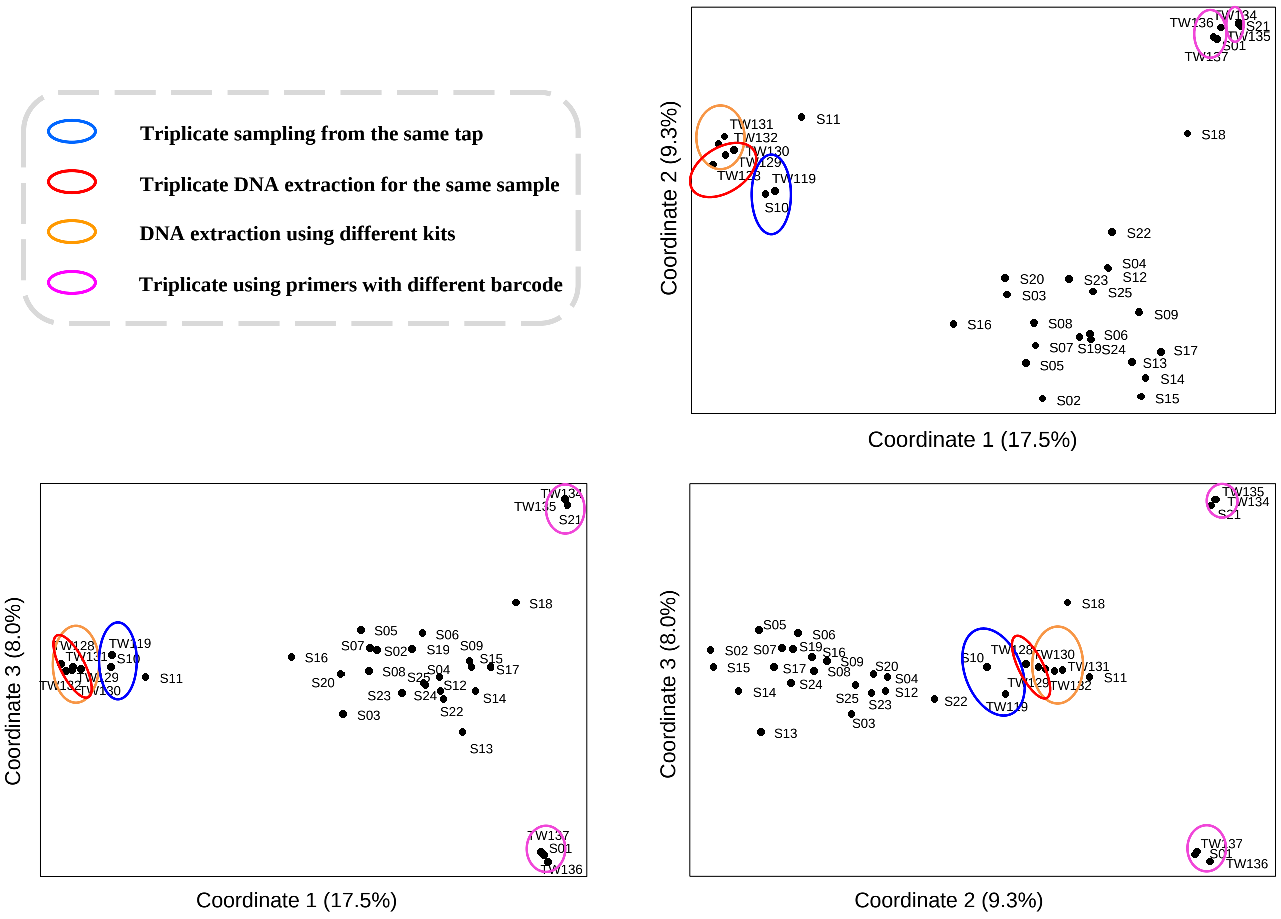

Fig. S6 Two-dimensional principal coordinate analysis plots show the microbial community difference among tap water samples. The analysis was conducted using the matrix of OTUs abundance (0.97 similarity) in tap water samples, and the weighted Unifrac algorithm was used to determine pairwise community distances.

References

1. Yang, Y., et al., *Exploring Variation of Antibiotic Resistance Genes in Activated Sludge over a Four-Year Period through a Metagenomic Approach.* Environmental Science & Technology, 2013. **47**(18): p. 10197-10205.
2. Kozich, J.J., et al., *Development of a dual-index sequencing strategy and curation pipeline for analyzing amplicon sequence data on the MiSeq Illumina sequencing platform.* Appl Environ Microbiol, 2013. **79**(17): p. 5112-20.
3. Edgar, R.C., et al., *UCHIME improves sensitivity and speed of chimera detection.* Bioinformatics, 2011. **27**(16): p. 2194-2200.
4. Caporaso, J.G., et al., *QIIME allows analysis of high-throughput community sequencing data.* Nature Methods, 2010. **7**(5): p. 335-336.
5. Junker, B. and F. Schreiber, *Correlation Networks. In. Analysis of Biological Networks.* Wiley-Interscience, 2008.
6. Newman, M.E.J., *Modularity and community structure in networks.* Proceedings of the National Academy of Sciences of the United States of America, 2006. **103**(23): p. 8577-8582.
7. Li, B., et al., *Metagenomic and network analysis reveal wide distribution and co-occurrence of environmental antibiotic resistance genes.* Isme Journal, 2015. **9**(11): p. 2490-2502.
8. Kohler, T., et al., *Characterization of MexE-MexF-OprN, a positively regulated multidrug efflux system of Pseudomonas aeruginosa.* Molecular Microbiology, 1997. **23**(2): p. 345-354.
9. Tosini, F., et al., *Class 1 integron-borne multiple-antibiotic resistance carried by IncFI and IncL/M plasmids in Salmonella enterica serotype typhimurium.* Antimicrobial Agents and Chemotherapy, 1998. **42**(12): p. 3053-3058.
10. Samanta, I., et al., *Prevalence and antibiotic resistance profiles of Salmonella serotypes isolated from backyard poultry flocks in West Bengal, India.* Journal of Applied Poultry Research, 2014. **23**(3): p. 536-545.
11. Ma, L.P., et al., *Metagenomic Assembly Reveals Hosts of Antibiotic Resistance Genes and the Shared Resistome in Pig, Chicken and Human Feces.* Environmental science & technology, 2016. **50**(1): p. 420-427.
